# Disrupted functional network integrity and flexibility after stroke: Relation to motor impairments

**DOI:** 10.1016/j.nicl.2018.06.010

**Published:** 2018-06-09

**Authors:** Sara Larivière, Nick S. Ward, Marie-Hélène Boudrias

**Affiliations:** a McConnell Brain Imaging Centre, Montreal Neurological Institute and Hospital, McGill University, Montréal, Qc, Canada; b Department of Neurology and Neurosurgery, McGill University, Montréal, Qc, Canada; c Sobell Department of Motor Neuroscience, Institute of Neurology, University College, London, UK; d School of Physical and Occupational Therapy, McGill University, Montréal, Qc, Canada; e Center for Interdisciplinary Research in Rehabilitation of Greater Montreal (CRIR), Montréal, Qc, Canada

**Keywords:** Stroke, Functional magnetic resonance imaging, Hand movement, Motor deficits, Network analysis, Network flexibility

## Abstract

Previous studies investigating brain activation present during upper limb movement after stroke have greatly detailed activity alterations in the ipsi- and contralesional primary motor cortices (M1). Despite considerable interest in M1, investigations into the integration and coordination of large-scale functional networks subserving motor, sensory, and cognitive control after stroke remain scarce. The purpose of this study was to assess non-static functional connectivity within whole-brain networks involved in the production of isometric, visually-paced hand grips. Seventeen stroke patients and 24 healthy controls underwent functional MRI while performing a series of 50 isometric hand grips with their affected hand (stroke patients) or dominant hand (control subjects). We used task-based multivariate functional connectivity to derive spatial and temporal information of whole-brain networks specifically underlying hand movement. This technique has the advantage of extracting within-network commonalities across groups and identifying connectivity differences between these groups. We further used a nonparametric statistical approach to identify group differences in regional activity within these task-specific networks and assess whether such alterations were related to the degree of motor impairment in stroke patients. Our whole-brain multivariate analysis revealed group differences in two networks: (1) a motor network, including pre- and postcentral gyri, dorsal and ventral premotor cortices, as well as supplementary motor area, in which stroke patients showed reduced task-related activation compared to controls, and (2) a default-mode network (DMN), including the posterior cingulate cortex, precuneus, and medial prefrontal cortex, in which patients showed less deactivation than controls. Within-network group differences revealed decreased activity in ipsilesional primary sensorimotor cortex in stroke patients, which also positively correlated with lower levels of motor impairment. Moreover, the temporal information extracted from the functional networks revealed that stroke patients did not show a reciprocal DMN deactivation peak following activation of their motor network. This finding suggests that allocation of functional resources to motor areas during hand movement may impair their ability to efficiently switch from one network to another. Taken together, our study expands our understanding of functional reorganization during motor recovery after a stroke, and suggests that modulation of ipsilesional sensorimotor activity may increase the integrity of a whole-brain motor network, contribute to better motor performance, and optimize network flexibility.

## Highlights

• We assessed connectivity in task-based brain networks underlying hand movement.
• We compared network properties between stroke patients and healthy controls.
• Stroke patients have altered default-mode and motor network connectivity.
• Patients also showed poorer motor performance and impaired network flexibility.

## 1 Introduction

Stroke is a cerebrovascular injury often resulting in sensorimotor and cognitive impairments (Broeks et al., 1999; Desmond et al., 1996). While some patients achieve good motor recovery, up to 40% of stroke survivors are left with permanent motor disabilities (Krueger et al., 2015). A key impediment to the development of effective treatment interventions lies in the lack of empirical evidence linking residual motor functions to observed functional connectivity changes in widely distributed regions outside the lesion site (Fornito et al., 2015). Stroke research grounded in network analysis is therefore crucial to understand the mechanisms that enable motor recovery after an infarct. Identification of network abnormalities in stroke patients can be leveraged using task-based functional neuroimaging paradigms, which, as opposed to resting-state (i.e., task-free) recordings, have the ability to detect brain alterations that may only manifest during the performance of motor actions. Despite considerable interest in the role of the primary motor cortex (M1) after a stroke, our knowledge on the integrative and cooperative properties of functionally connected, large-scale brain networks involved in the execution of hand movement in stroke patients remain scarce (Bernhardt et al., 2016; Rehme et al., 2015).

Recent developments in the field of network neuroscience have revealed that an optimal brain requires a dynamic and flexible balance between unimodal (e.g., sensorimotor, visual) and transmodal (e.g., attention, default-mode) large-scale networks (Bressler and Menon, 2010; Margulies et al., 2016). Network flexibility, which reflects the brain's ability to switch between different network configurations, has been demonstrated to change dynamically during a simple motor learning task, balancing between attention- and motor-driven processes (Bassett et al., 2011). Efforts to characterize motor recovery mechanisms in stroke individuals, however, have focused almost exclusively on static patterns of functional connectivity in individual networks. Several lines of research comparing stroke patients to healthy controls, for instance, have reported decreased interhemispheric motor network connectivity (Park et al., 2011; Thiel and Vahdat, 2015), disruption of the dorsal attention network (Carter et al., 2010; He et al., 2007), and an inability to regulate default-mode network (DMN) activity (Dacosta-Aguayo et al., 2015; Tuladhar et al., 2013). Although the majority of these findings correlated with behavioral measures of cognition, attention, and motor impairment, these observations also raise the possibility that connectivity alterations in diverse cerebral systems may lead to impaired network flexibility after a stroke.

A large proportion of the studies on stroke have used univariate analysis methods which limit the observations of brain activity to individual regions that are largely independent of each other. However, because motor and cognitive networks are active in parallel during a hand motor task (Bressler and Menon, 2010), univariate task-based regression methods may lead researchers to overlook important information embedded in the functional integrity and cooperation of large-scale networks. In contrast, multivariate methods can separate multiple distinct, simultaneously active brain networks while quantifying each network's unique spatial and temporal properties. In this study, we derived task-specific functional brain networks with subject- and condition-specific hemodynamic response (HDR) shapes using constrained principal component analysis for functional MRI (fMRI-CPCA; www.nitrc.org/projects/fmricpca), a method that integrates multivariate multiple regression analysis and principal component analysis into a unified framework. Notably, fMRI-CPCA has the advantage of identifying brain networks that are (1) specifically underlying isometric hand grips, and (2) shared across all subjects, thus allowing direct comparison of network connectivity between groups. Previous studies employing similar approaches to examine whole-brain patterns of functional connectivity have reported a progressive increase in motor network connectivity and integrity during the recovery process after stroke (Wadden et al., 2015; Wang et al., 2010). While these studies have documented connectivity anomalies extending beyond individual motor regions, the consequences of stroke on functional integrity and coordination between motor and cognitive networks remain to be investigated.

Our purpose was to compare non-static functional connectivity changes in whole-brain networks between stroke patients and healthy controls during the production of isometric hand grips. We used fMRI-CPCA to generate shared functional networks that activate (or deactivate) during hand movement and extract each network's spatial and temporal patterns of activation. In line with previous studies highlighting regional activity decreases in several functionally segregated motor areas (for comprehensive reviews, see Lake et al. (2016) and Grefkes and Fink (2011)), we hypothesized that patients would show reduced whole-brain motor network functional connectivity relative to controls. Furthermore, based on evidence from resting-state and hand motor task studies suggesting altered activity in sensorimotor and default-mode regions (Dacosta-Aguayo et al., 2015; Liu et al., 2014; Wang et al., 2010; Ward et al., 2003), we postulated that differential patterns of network connectivity in stroke patients may lead to impaired network flexibility. In light of the notion that reinstatement of previously reduced functional activations can predict post-stroke motor recovery (Kim and Winstein, 2017), we also assessed the relationship between regional brain activity changes and variability in behavioral motor performance in stroke patients.

## 2 Materials and methods

### 2.1 Participants

A total of 41 subjects (17 stroke patients and 24 healthy controls) were included in this study. All patients had suffered from first ischemic stroke; individual patient characteristics are presented in Supplementary Table 1. Group-specific demographic information is listed in Table 1; groups were matched on gender, handedness, and age. Full written consent was obtained from all subjects in accordance with the Declaration of Helsinki. The study was approved by the Joint Ethics Committee of the Institute of Neurology, UCL and NHNN, UCL Hospitals NHS Foundation Trust, London.

**Table 1:** Participants' demographic information and behavioral scores. *Note: standard deviations are in parentheses. BBT, Box and Block Test; NHPT, Nine-Hole Peg Test.

Variable	Control subjects	Stroke patients
Sex (male/female)	14/10	14/3
Handedness (right/left)	23/1	17/0
Age (years)	46.7 (17.5)	53.2 (12.3)
Time since stroke (months)	–	44.9 (56.6)
Lesion side (right/left)	–	11/6
Hand affected (right/left)	–	6/11
BBT % of unaffected	–	52.1 (26.6)
NHPT % of unaffected	–	40.8 (35.5)
Grip strength % of unaffected	–	56.0 (33.7)

### 2.2 Experiment protocol

#### 2.2.1 Behavioral assessment

Motor impairment was assessed based on measurements of (1) hand grip strength (Mathiowetz et al., 1984), (2) finger dexterity (Nine Hole Peg Test; Mathiowetz et al., 1984), and (3) unilateral gross manual dexterity (Box and Block Test; Mathiowetz et al., 1985). As depicted in Table 1, these measurements were calculated as a percentage of the score obtained with the unimpaired hand (Sunderland et al., 1989). These scores were then entered into a principal component analysis (PCA) and the first component was used as a single impairment score for each patient, with lower motor score values corresponding to greater motor impairment.

#### 2.2.2 Motor task

While undergoing fMRI, all subjects performed a series of 50 visually cued isometric hand grips, using an MR-compatible manipulandum as described elsewhere (Ward and Frackowiak, 2003). Healthy controls carried out the task with their dominant hand while patients performed the task with their affected (i.e., contralesional) hand. Each subject performed a total of 50 isometric hand grips at a target pressure of 10% or 30% of their maximum voluntary contraction in a randomized order. Hand grips were sustained for 3 s and were followed by a variable interstimulus interval between 3 and 7 s.

### 2.3 Data analysis and functional connectivity

Details regarding data acquisition and preprocessing are described in the Supplementary Material. To allow for direct comparison between groups, images from the right-sided stroke patients (*n* = 11) were flipped about the midsagittal plane so that the lesioned hemisphere corresponded to the left hemisphere. Data from the left-handed control subject (*n* = 1) were also flipped so to conform to the rest of the control group (i.e., a left-dominant hemisphere).

The data were analyzed using fMRI-CPCA with orthogonal rotation (Metzak et al., 2011; Takane and Hunter, 2001; Woodward et al., 2013). Briefly, fMRI-CPCA integrates multivariate multiple regression analysis and PCA into a unified framework. This method enables derivation of brain networks from variations of the task-related blood oxygen level-dependent (BOLD) signal, but also allows for identification of functional brain networks that vary as a function of task-timing. As opposed to univariate methods, in which BOLD responses in each brain voxel are analyzed independently, fMRI-CPCA allows for the analysis of functionally connected networks of brain regions, and identification of their role in specific cognitive and motor processes as they occur over poststimulus time for different groups. Brain networks are isolated by performing a PCA on the task-related variance in brain activity, which results in independent sources of variance reflecting task-specific brain networks. In the current study, we regressed out the rigid-body parameters prior to other task-unrelated variance. We used a finite impulse response model; the six poststimulus time points correspond to the 1st to 6th full brain scans following stimulus presentation. The repetition time for these data was 3.25 s, which resulted in an estimated BOLD signal over a 19.5 s time period, with the first time point (time = 0) corresponding to stimulus onset.

### 2.4 Statistical analysis

The cognitive and motor functions of each brain network were interpreted by analyzing predictor weights that produce subject- and condition-specific estimated HDR shapes. Specifically, these predictor weights were applied to the finite impulse response model used in the current analysis. The resulting functional brain networks were then interpreted spatially by examining the dominant patterns of intercorrelated voxels, and temporally by looking at their associated HDR shapes. Predictor weights producing the HDR shapes were submitted to statistical analyses to test whether each functional network reflected a reliable hemodynamic response as well as to test differences in activation of each functional network between conditions and between groups (Larivière et al., 2017; Lavigne et al., 2015). These analyses were examined using four 6 × 2 × 2 mixed-model ANOVAs (four components extracted; see Results), with the within-subjects factors of Poststimulus Time (6 poststimulus time points) and Force (10%, 30%), and the between-subjects factor of Group (controls, stroke patients). Tests of sphericity were carried out for all ANOVAs and Greenhouse-Geisser adjusted degrees of freedom were checked. Original degrees of freedom are reported here as violations of sphericity did not affect the results. Post hoc findings were corrected for multiple comparisons, controlling at a false discovery rate (FDR) of *p*_*FDR*_ < 0.05 (Benjamini and Hochberg, 1995). To illustrate trends, we also displayed uncorrected findings.

### 2.5 Within-network analysis

We used a nonparametric statistical method (e.g., FSL's Randomise permutation-testing tool, run with 5000 permutations) to investigate group differences within the identified task-specific functional brain networks. Activity differences between controls and stroke patients were constrained to the extreme 10% of voxels (i.e., highest component loadings) for each shared functional network from the fMRI-CPCA output. As such, differences in activation of individual brain regions were examined within the data-driven, task-based brain networks, thus avoiding potential bias that may arise from choosing a priori regions of interest. Significant group differences were identified using threshold-free cluster enhancement and were corrected for multiple comparisons using family-wise error (Smith and Nichols, 2009).

## 3 Results

### 3.1 Lesion overlap

**Fig. 1:**
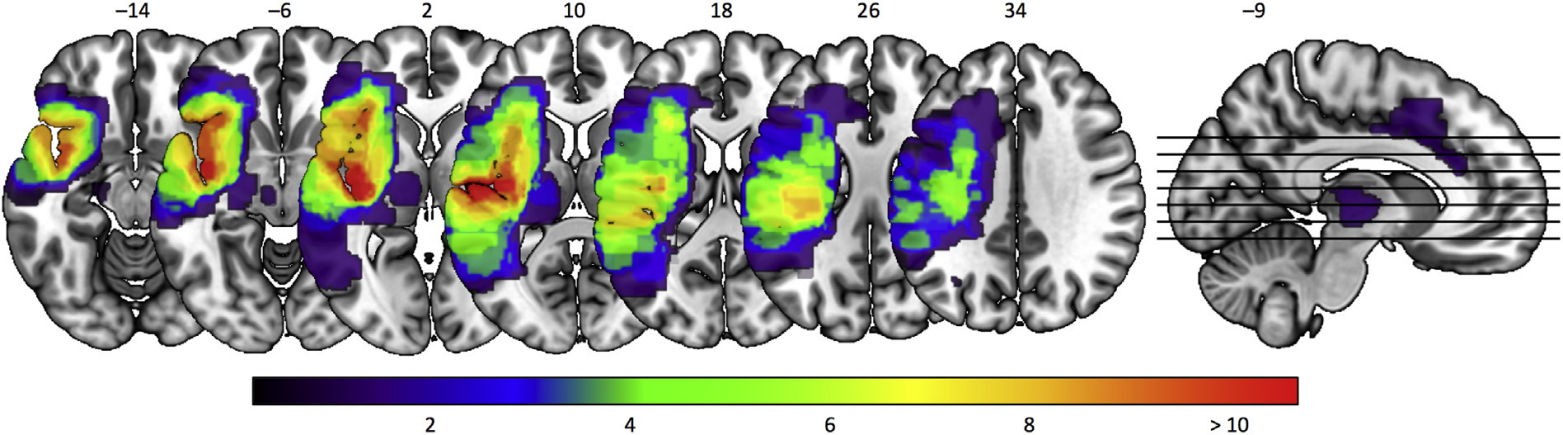
Lesion locations in all stroke patients. The heatmap represents the degree of overlap, with the purple end of the spectrum indicating voxels damaged in one patient, and shades of red indicating voxels damaged in a larger number of patients.

The brain lesions of all 17 stroke patients are displayed in Fig. 1 (superimposed on the brain image). The lesions' overlap was found mainly along the corticospinal tract at the level of the internal capsule, as well as in the insula, ventral striatum, parietal and central operculum cortices, precentral gyrus, temporal pole, inferior frontal gyrus, and supramarginal gyrus. To assess the relation between lesion location and our connectivity findings, we computed Dice similarity coefficients, a quantitative index of spatial overlap ranging between 0 and 1, between the lesion overlap map and the dominant 10% of each identified task-based brain networks (see section 3.3). Dice indices for all identified networks were below 0.097, indicating minimal spatial overlap with the lesion overlap map.

### 3.2 Behavioral results

For the stroke group, the percentage of variance for the first principal component of the three motor scores was 82.2% and was therefore used as the representative motor impairment score. A lower principal component score represents greater motor impairment. Comparison of raw motor performance scores for controls and stroke patients can be found in Supplementary Table 2.

### 3.3 Functional connectivity

**Fig. 2:**
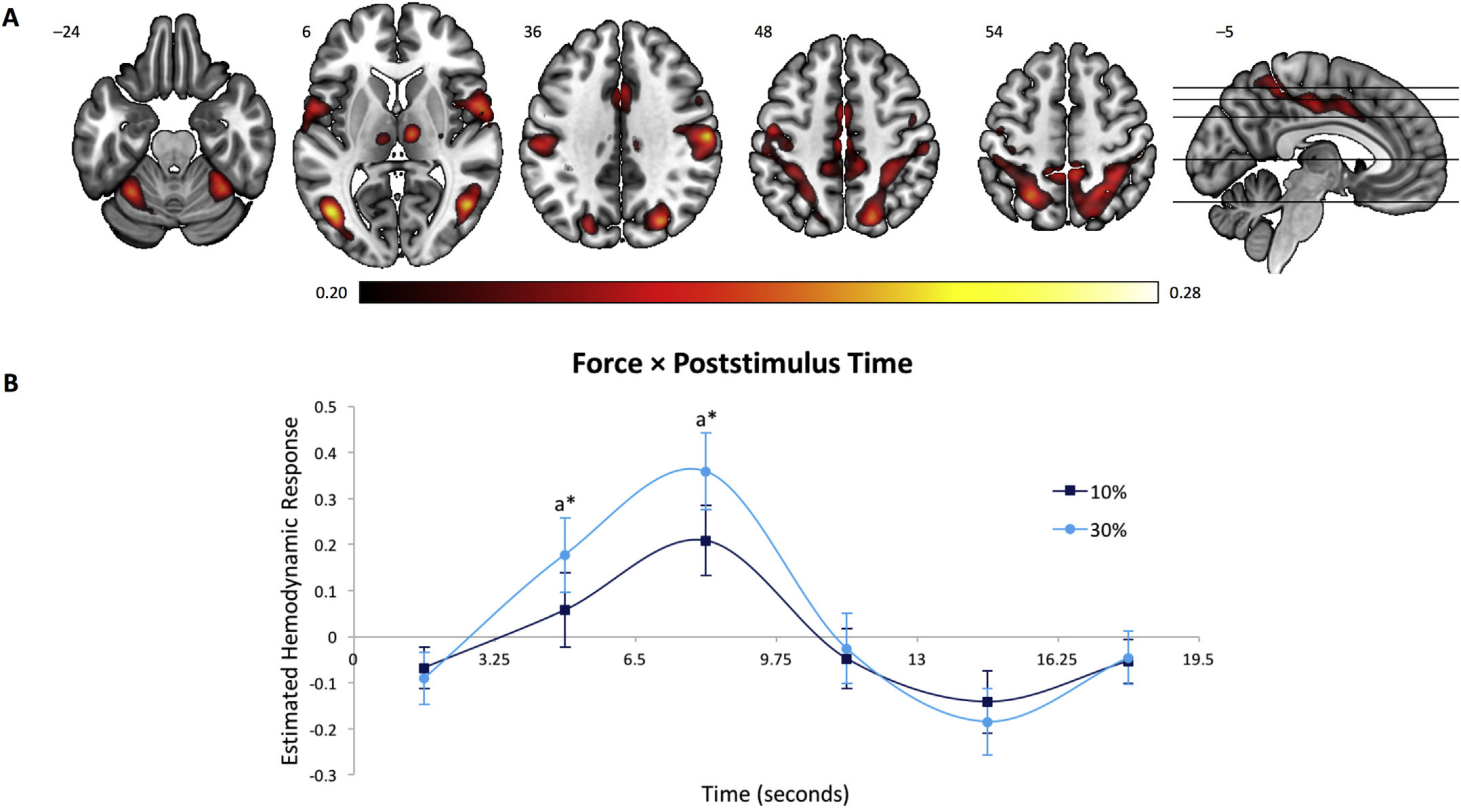
Brain regions and estimated HDR associated with the Dorsal Attention Network. (A) Dominant 5% of component loadings for the Dorsal Attention Network (Network 1); positive loadings in red, threshold = 0.20, max = 0.28, no negative loadings. Montreal Neurological Institute *Z*-axis coordinates are displayed. (B) Mean finite impulse response-based predictor weights averaged across groups, plotted as a function of poststimulus time. ^a^ = 30% > 10%. ^⁎^ = *p*_FDR_ < 0.05. Error bars are standard errors.

**Fig. 3:**
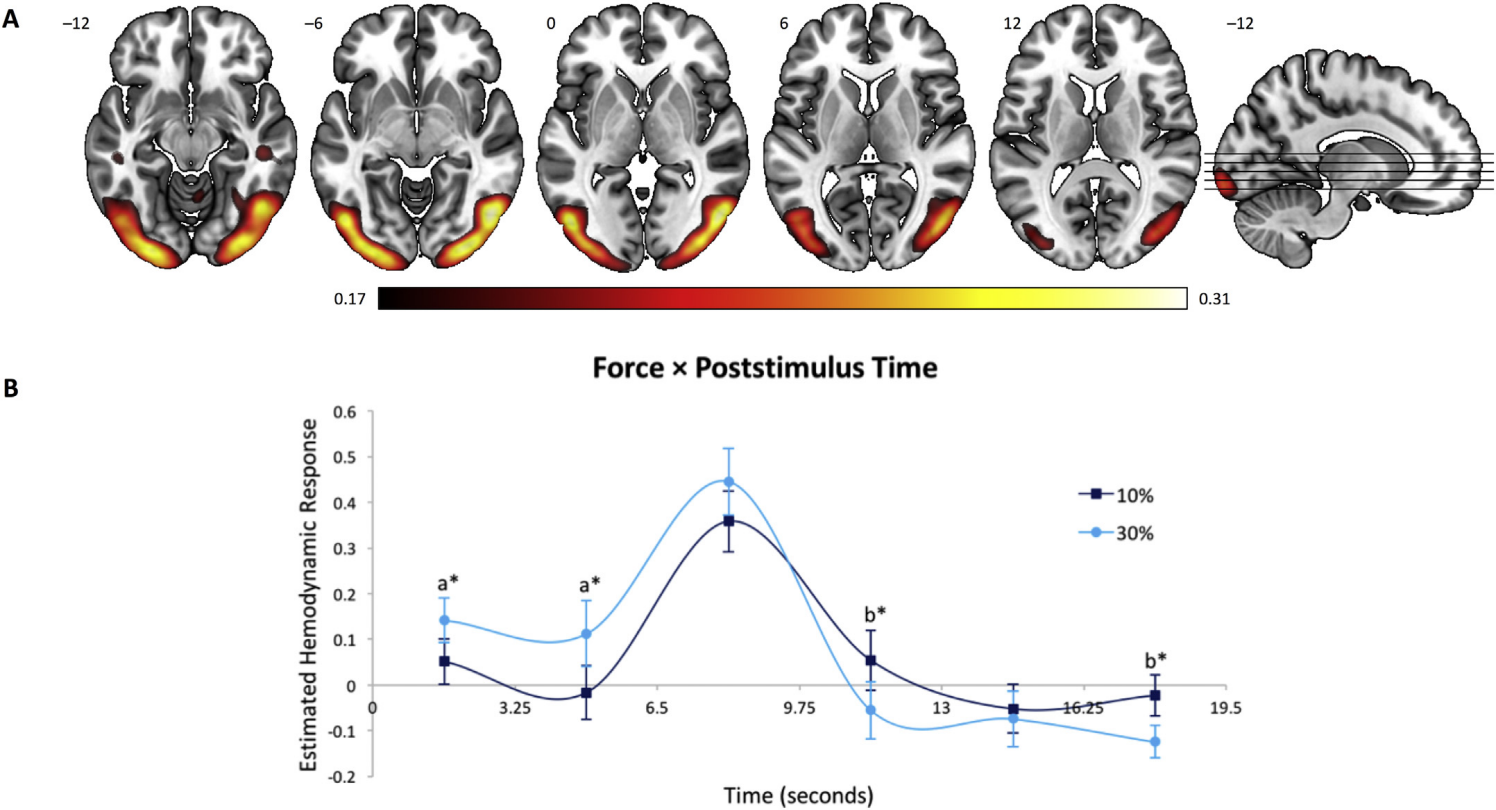
Brain regions and estimated HDR associated with the Visual Network. (A) Dominant 5% of component loadings for the Visual Network (Network 2); positive loadings in red, threshold = 0.17, max = 0.31, no negative loadings. Montreal Neurological Institute Z-axis coordinates are displayed. (B) Mean finite impulse response-based predictor weights averaged across groups, plotted as a function of poststimulus time. ^a^ = 30% > 10%; ^b^ = 10% > 30%. ^⁎^ = *p*s < 0.05, uncorrected. Error bars are standard errors.

**Fig. 4:**
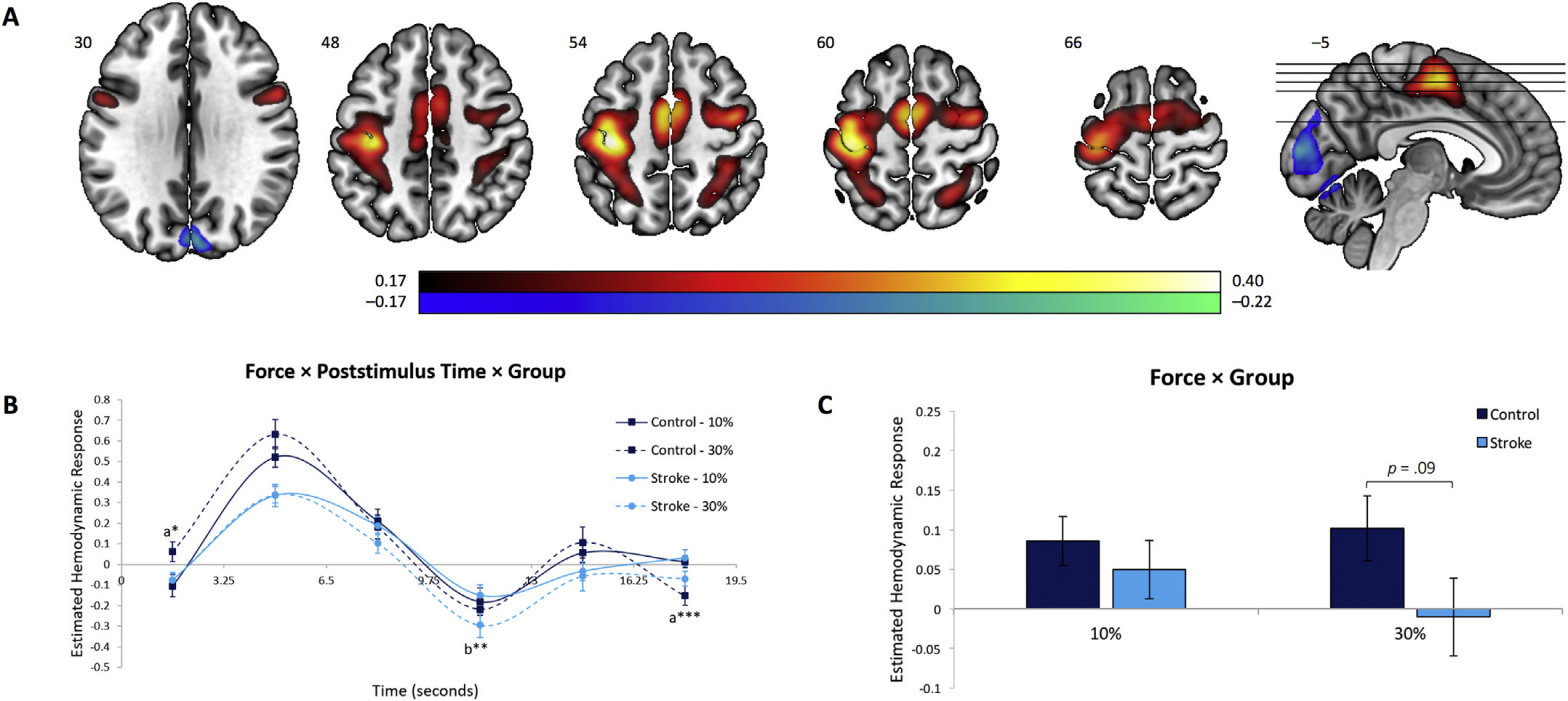
Brain regions and estimated HDR associated with the Motor Network. (A) Dominant 5% of component loadings for the Motor Network (Network 3); positive loadings in red, negative loadings in blue, threshold = ±0.17, min = −0.22, max = 0.40. Montreal Neurological Institute Z-axis coordinates are displayed. (B) Mean finite impulse response-based predictor weights averaged across all time points, plotted as a function of condition. (C) Mean finite impulse response-based predictor weights for each combination of group and condition, plotted as a function of poststimulus time. ^a^ = 30% > 10%; ^b^ = 10% > 30%. ^⁎^ = *p* < 0.05, uncorrected; ^⁎⁎^ = *p*_FDR_ < 0.05; ^⁎⁎⁎^ = *p*_FDR_ < 0.001. Error bars are standard errors.

**Fig. 5:**
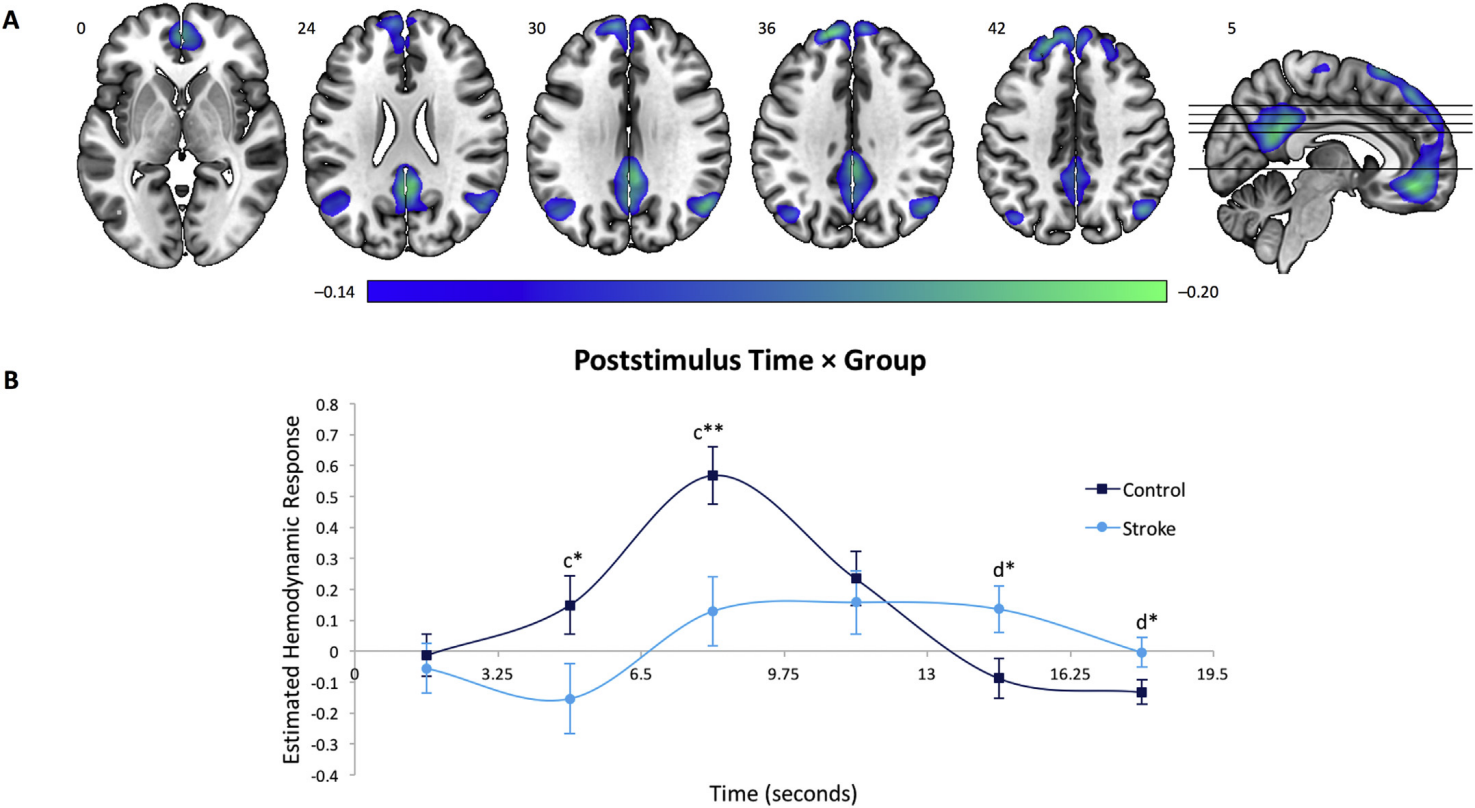
Brain regions and estimated HDR associated with the Default-Mode Network. (A) Dominant 5% of component loadings for the Default-Mode Network (Network 4); negative loadings in blue, threshold = −0.14, min = −0.20, no positive loadings. Montreal Neurological Institute Z-axis coordinates are displayed. (B) Mean finite impulse response-based predictor weights averaged across conditions, plotted as a function of poststimulus time. ^c^ = Control > Stroke; ^d^ = Stroke > Control. ^⁎^ = *p* < 0.05, uncorrected; ^⁎⁎^ = *p*_FDR_ < 0.05. Error bars are standard errors.

The scree plot of singular values revealed four predominant components (i.e., networks shared across all subjects) accounting for task-related variance in brain activity. The percentages of task-related variance were 12.1%, 6.7%, 6.3%, and 4.5% for networks 1–4, respectively. The brain regions associated with networks 1, 2, 3, and 4 are displayed in Fig. 2, Fig. 3, Fig. 4, Fig. 5, respectively, with estimated HDR shape of each functional network represented by predictor weights plotted as a function of poststimulus time. Anatomical descriptions for each component are presented in Supplementary Tables 3–6.

#### 3.3.1 Network 1: dorsal attention network

This network (Fig. 2) was characterized by bilateral activations in regions associated with the dorsal attention network (Yeo et al., 2011), namely inferior frontal gyrus, pars opercularis, anterior intraparietal sulcus, inferior and superior parietal lobules, as well inferior and middle temporal gyri. Activity increases were also observed in the anterior cingulate cortex and cerebellum. Predictor weights reflecting the estimated HDR for Network 1 were submitted to a mixed-model ANOVA; we found a significant main effect of Poststimulus Time, *F*_5,195_ = 9.31, *p* < 0.001, η^2^_p_ = 0.19, indicating that this network reflects a reliable HDR shape as opposed to varying randomly around zero. A significant Force × Poststimulus Time interaction was also observed, *F*_5,195_ = 2.49, *p* < 0.05, η^2^_p_ = 0.06, and a follow up analysis of simple main effects revealed a distinctly higher peak (at 4.9 and 8.1 s; *p*_FDR_ < 0.05) in the 30% relative to the 10% force condition (Fig. 2B). No significant differences between groups emerged (*p*s > 0.10).

#### 3.3.2 Network 2: visual network

This network (Fig. 3) was characterized by bilateral activations in primary visual network and extending laterally into the secondary visual areas, extrastriate cortex, as well as ventrally into the inferior temporal cortex. Predictor weights reflecting the estimated HDR for Network 2 were submitted to a mixed-model ANOVA. As for the Dorsal Attention Network (Network 1), this network showed a significant main effect of Poststimulus Time, *F*_5,195_ = 15.54, *p* < 0.001, η^2^_p_ = 0.28, and Force × Poststimulus Time interaction, *F*_5,195_ = 5.77, *p* < 0.001, η^2^_p_ = 0.13. A subsequent analysis of simple main effects indicated that this interaction was near significant at 1.6, 4.9, 11.4, and 17.9 s (*p*_FDR_ = 0.06, *p*s < 0.05, uncorrected), reflecting a slightly earlier and higher HDR shape in the 30% condition relative to the 10% force condition (Fig. 3B). No significant main effects or interactions involving Group were observed (*p*s > 0.55).

#### 3.3.3 Network 3: motor network

This network (Fig. 4) was largely dominated by left-lateralized activations in motor regions, specifically M1, supplementary motor area (SMA), posterior parietal cortex, as well as ventral and dorsal premotor cortices (PMv, PMd). The spatial distribution of this network is reflective of sensorimotor response processes involved in isometric right-hand grips. This network was also characterized by BOLD signal decreases bilaterally in the primary visual cortex. Based on a mixed-model ANOVA, predictor weights reflecting the estimated HDR for Network 3 showed a significant main effect of Poststimulus Time, *F*_5,195_ = 39.61, *p* < 0.001, η^2^_p_ = 0.50, as well as a significant Force × Poststimulus Time, *F*_5,195_ = 6.68, *p* < 0.001, η^2^_p_ = 0.15, and Force × Group, *F*_1,39_ = 5.13, *p* < 0.05, η^2^_p_ = 0.12. Follow up analyses of simple main effects revealed a non-significant trend towards a decrease in functional connectivity in regions comprising the motor network in stroke patients relative to control subjects in the 30% force condition (*p* = 0.09, uncorrected; Fig. 4B and C). This indicates that relative to controls, the brain network involved in the performance of hand motor movements in stroke patients is characterized by an overall BOLD response with lower peak magnitude and greater poststimulus undershoot.

#### 3.3.4 Network 4: default-mode network

This network (Fig. 5) was primarily characterized by BOLD signal decreases (i.e., deactivation) in regions associated with the well-documented DMN (Buckner et al., 2008; Raichle et al., 2001), notably in posterior cingulate cortex, precuneus, and medial prefrontal cortex. Statistical analysis of the predictor weights for Network 4 was carried out using a mixed-model ANOVA, and a significant main effect of Poststimulus Time, *F*_5,195_ = 12.43, *p* < 0.001, η^2^_p_ = 0.24, as well as a significant Poststimulus Time × Group interaction, *F*_5,195_ = 7.53, *p* < 0.001, η^2^_p_ = 0.16, were found. A subsequent analysis of simple main effects revealed that this interaction was strongest at 8.1 s (*p*_FDR_ < 0.05), reflecting a significantly higher deactivation peak in the control group relative to the stroke one (Fig. 5B). No significant differences emerged between the 10% and 30% force conditions (*p* > 0.90).

### 3.4 Within-network activity differences

**Fig. 6:**
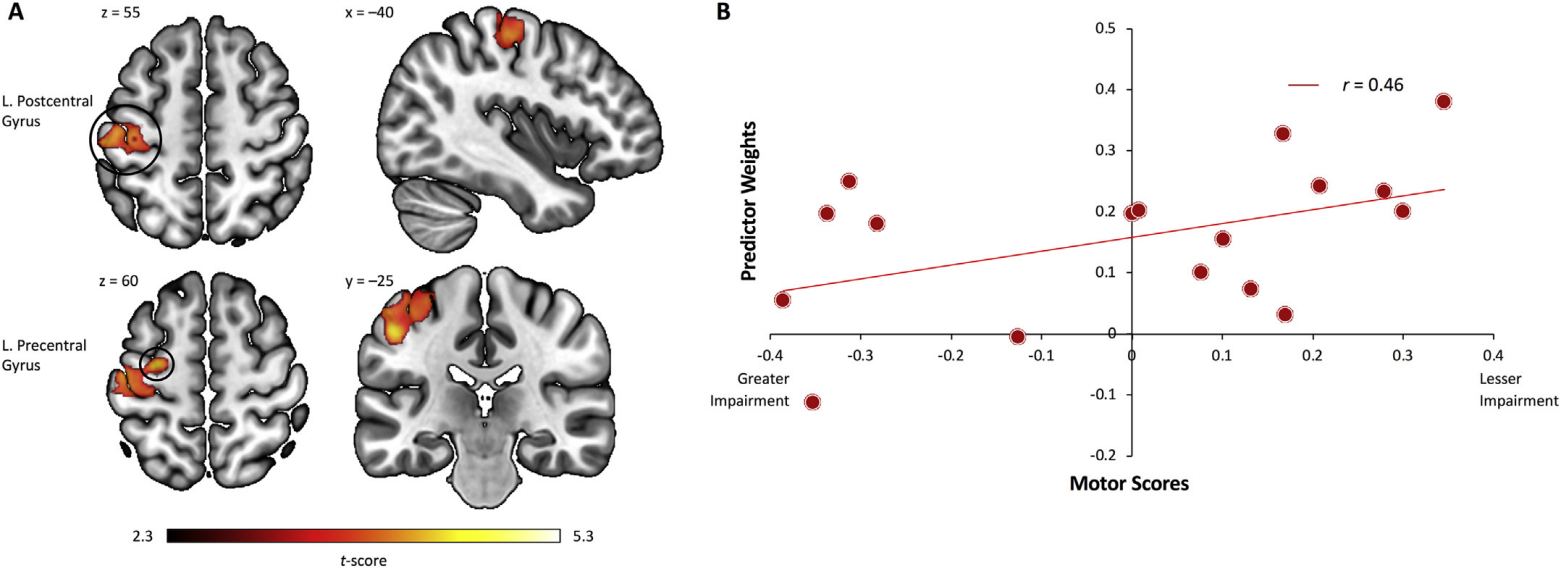
Within-network regional activity differences and relationship to motor performance. (A) The within-network analysis masked for the dominant 10% of component loadings for the Motor Network (Network 3) revealed significantly reduced activity in left sensorimotor regions (pre- and postcentral gyri) in stroke patients relative to control subjects (*p*_corr_ < 0.01). (B) Positive relationship between left sensorimotor activity and behavioral motor scores in stroke patients (*p* < 0.05).

Activation differences within the task-based brain networks derived from fMRI-CPCA were assessed using nonparametric permutation testing. This analysis yielded two significantly distinct clusters of voxels that were different between groups: (1) when masked for the motor network (Component 3), controls showed increased activation in a left M1/S1 cluster relative to stroke patients (Fig. 6A; *p*_corr_ < 0.01); and (2) when masked for the DMN (Component 4), controls showed increased bilateral precuneus deactivation relative to patients (Supplementary Fig. 1; *p* < 0.05).

### 3.5 Relationship between regional activity and motor performance

The relationship between regional brain activity and residual motor performance in stroke patients was assessed by computing data-driven correlations between the predictor weights of the within-network clusters (ipsilesional M1/S1 and precuneus) and the behavioral motor impairment scores (i.e., principal component scores). We found a significant negative relationship between ipsilesional pre- and postcentral gyri activation and motor impairment scores, *r* = −0.46, *p* < 0.05 (Fig. 6B). Since higher principal component scores equate greater motor impairment, increased brain activity within the sensorimotor cluster was associated with better motor performance. No significant correlations were found between motor performance and precuneus deactivations, as well as between functional connectivity within each whole-brain network, age, post-stroke duration, and lesion size (*p*s > 0.15).

## 4 Discussion

The goal of this study was to investigate functional connectivity alterations in brain networks underlying isometric, visually-paced hand grips in stroke patients relative to control subjects. Of the four functional brain networks identified, group differences were only observed in the motor network and the DMN, in which stroke patients expressed decreased functional connectivity relative to control subjects. Interestingly, as opposed to healthy controls, stroke patients did not show a reciprocal DMN deactivation peak following motor network activation, suggesting an impaired ability to efficiently switch from one network to another following initiation of the motor network. Additionally, activation of individual brain regions within the identified task-related motor network was reduced in the ipsilesional (contralateral to the hand) M1/S1 of stroke patients. Notably, decreased task-related activity within this region was associated with greater motor impairment. No group differences were observed in the dorsal attention and visual networks, however, overall increased activity in these networks was associated with production of higher force levels in all subjects. Collectively, these findings suggest that reduced regional brain activity in ipsilesional M1/S1 affects the efficacy of the motor network during the performance of hand movement, and impairs functional network flexibility in individuals with stroke.

### 4.1 Residual motor network connectivity and its relationship to behavioral impairments

Consistent with existing findings reporting activity reductions in various motor-related regions at rest and during a hand motor task (Pineiro et al., 2002; Wadden et al., 2015; Wang et al., 2010), we observed activity decreases in a whole-brain motor network in stroke patients relative to control subjects. Notably, with respect to behavioral performance we found that activity intensity within the ipsilesional M1/S1 cluster of the task-specific motor network was positively correlated with lower levels of motor impairment, thus representing a potential biomarker of residual motor performance after stroke. Interestingly, Borich et al. (2014) reported that a certain amount of residual corticospinal tract integrity must be preserved in stroke patients in order to observe meaningful behavioral motor performance changes following motor learning training after stroke. In line with this research, our within-network finding of reduced M1/S1 activity in patients with greater motor impairment may reflect underlying damage to corticospinal tract fibers originating from the ipsilesional hemisphere. Taken together, these findings bear important implications for stroke recovery rehabilitation; currently employed treatment approaches aiming to facilitate M1 excitability may not be beneficial for a substantial proportion of patients characterized with high degree of M1/S1 alterations. Our results rather suggest that secondary motor areas (e.g., bilateral PMv, PMd, and SMA), alongside bilateral parietal cortices, also strongly contribute to the task-specific motor network (as indexed by the lighter shades of red/white superimposed on the brain image in Fig. 4A). These regions seem to contribute to a brain circuit that becomes critically important to support residual motor function, and which allow more impaired patients to perform hand movements by increasing their contribution in terms of motor outputs to the spinal cord where motoneurons are located (Boudrias et al., 2009a; Boudrias et al., 2009b).

### 4.2 Connectivity changes in higher-order functional networks

There is compiling evidence suggesting that brain lesions can disrupt connectivity in large-scale networks subserving higher-order functioning (Corbetta et al., 2005; Dacosta-Aguayo et al., 2015; Tuladhar et al., 2013). One such network, the dorsal attention network, has been consistently shown to be activated during attention-demanding tasks (Corbetta and Shulman, 2002; Spreng et al., 2013). A longitudinal study on stroke patients presenting attentional deficits (i.e., visuospatial neglect; Nijboer et al., 2013) showed that functional connectivity within the dorsal attention network was highly disrupted during the acute stage post-infarct (~1 month) but was fully recovered in the chronic stage (>6 months; He et al., 2007). Similarly, when compared to controls, we found no breakdown of functional connectivity in the dorsal attention network in the stroke group, suggesting that alterations within this network may be specific to the pathophysiology of neglect during the acute stage after a stroke. Despite significant impairments in motor performance and motor connectivity, we observed that stroke patients maintained the ability to regulate activity of the attention network while increasing the level of force produced. As opposed to the externally-oriented dorsal attention network, the DMN has been predominantly associated with self-generated thoughts and mind wandering (Buckner et al., 2008; Gusnard and Raichle, 2001; Mason et al., 2007; Raichle et al., 2001). In an elegant paper by Margulies et al. (2016), hierarchical organization of large-scale connectivity in healthy adults was described by means of connectivity gradients, which reflect spatial differences in connectivity profiles (Coifman et al., 2005). The authors concluded that the DMN and primary sensory networks (e.g., sensorimotor, visual, and auditory) were anchored on opposite ends of a connectivity gradient spectrum, thus providing evidence that the DMN may play a functional role during tasks that require the integration of information from multiple sensory systems (Margulies et al., 2016). In line with this theory, the inability of stroke patients to deactivate the DMN, as observed in the current study and elsewhere (Liu et al., 2014; Park et al., 2014; Tuladhar et al., 2013), may reflect disruptions within the sensorimotor to higher-order connectivity gradient. One hypothesis is that the DMN, being located at the top of a representational hierarchy, recognizes hypoactivity of the motor network and consequently engages its main hubs (e.g., precuneus, medial prefrontal cortex) in an attempt to support residual motor function. Alternatively, the absence of a reciprocal DMN deactivation peak following motor network activation observed in stroke patients may provide evidence that network flexibility, hereby referring to the ability of brain networks to switch from unimodal (i.e., sensorimotor) to transmodal (i.e., default-mode) networks, becomes impaired after an infarct. In favor of the latter hypothesis, we did not find an association between DMN activity (or precuneus activity alone) and motor performance, thus suggesting that allocation of resources in stroke patients may be preferentially devoted to activation of motor areas, which in turn hinders network flexibility. Additional analyses splitting our stroke patient cohort into highly impaired patients (*n* = 12) and control patients (i.e., those who retained at least 70% of motor function relative to their unaffected hand, *n* = 5) further revealed an apparent decrease in network flexibility in patients with greater motor impairment relative to control patients. It is therefore unlikely that the group differences observed in the DMN reflect the general stroke effect, however, future studies are needed to better characterize the dynamic interplay of functional networks involved in the reorganization of brain networks associated with residual hand movement and recovery of functions.

### 4.3 Neurovascular alterations in stroke patients

The association between motor performance and underlying brain activity in the motor network may provide insights into long-term neurovascular alterations known to be present in stroke patients (Yanev and Dijkhuizen, 2012). In fact, increases in BOLD response, commonly interpreted as an indirect measure of neural activity, are driven by simultaneous changes in three factors, namely: cerebral blood flow cerebral blood volume, and metabolic rate of oxygen consumption (Buxton et al., 2004; Goense and Logothetis, 2008). Interestingly, multimodal studies using magnetoencephalography and fMRI in chronic stroke patients with good motor recovery have shown that absent or reduced of BOLD activity may not necessarily indicate an absence of neuronal activity but may instead reflect altered cerebral hemodynamics such as a decreased in cerebral blood flow (Altamura et al., 2007; Rossini et al., 2004). Alternatively, the lack of concordance between the results obtained from these two modalities could be due to the use of univariate voxelwise fMRI analyses, which may not be sensitive enough to detect task-specific BOLD alterations (Rowe, 2010). Here, we estimated the BOLD response underlying hand movement using a finite impulse response model which, unlike typical hemodynamic response function models, does not require any a priori assumption concerning the shape of the HDR (Henson et al., 2001). Consequently, this technique allowed the quantification of the primary BOLD response as well as the poststimulus undershoot (i.e., BOLD signal reduction below baseline). Whereas the former is classically characterized as neural activity, it has been hypothesized that poststimulus undershoots reflect concurrent reductions in neural activity, cerebral blood flow, and changes in cerebral blood volume (Mullinger et al., 2013). Although there were no significant differences between patients and controls at the peak of the HDR shape (i.e., primary BOLD response) in the motor network, stroke patients demonstrated a larger and wider poststimulus undershoot when producing higher level of force. Using pulsed arterial spin labeling, Brumm et al. (2010) found that cerebral blood flow was significantly reduced in anatomically intact regions in chronic stroke survivors. In line with this finding, we can speculate that modulation of grip force in stroke-impaired patients targets suboptimal neurovascular mechanisms within the motor network. Quantification of motor connectivity using a model-free approach (e.g., finite impulse response) therefore better characterizes underlying diffuse cerebral vascular dysregulations in the ischemic brain (Pineiro et al., 2002). Further investigation of the effects of stroke on the biological basis of the BOLD signal as well as the long-term neurovascular consequences of an ischemic lesion could become instrumental in neuroimaging research of cerebrovascular patients.

### 4.4 Limitations and future directions

The four brain networks derived from our multivariate functional connectivity analysis accounted for approximately one-third of task-related variance. It is therefore possible that the remaining sources of variance may explain subject-specific functional recovery processes, which our group-level analysis was unable to detect due to the inter-subject variability present in brain activation patterns in the stroke group. Arguably, our findings could be hampered by the large variability in post-stroke recovery phase (i.e., time since stroke), however introducing the covariate ‘time since stroke’ in our analysis did not alter the results. Despite that, we cannot absolutely exclude the influence ‘time after stroke’ on functional connectivity alterations. In view of these limitations, studies may wish to longitudinally track whole-brain functional reorganization after a stroke, from acute to chronic stages.

### 4.5 Conclusion

In summary, the ability to regulate activity of the motor network, notably within ipsilesional sensorimotor regions seems to play a crucial role in the recovery of motor functions observed in stroke patients. The overall motor network connectivity decreases observed in stroke patients appear to be driven by significant alterations in ipsilesional M1/S1. The fact that secondary motor areas such as SMA, PMv, and PMd were functionally intact suggest that they play a key role in supporting residual motor function after an infarct. Moreover, allocation of functional activations in motor areas during hand movement appeared to impair the ability of stroke patients to efficiently switch from the motor network to the DMN. In addition to quantifying the brain's functional networks involved in hand movement, our whole-brain, task-based functional connectivity analysis lends a foundation that could allow future multimodal studies to integrate non-static properties of brain networks with changes in vascular health in at-risk populations. Taken together, our study highlights abnormal timing and activation patterns in large-scale motor and default-mode networks, and further establishes ipsilesional sensorimotor regions as an important biological marker of the motor state in stroke patients. Modulation of activity within these motor regions may therefore increase the integrity of whole-brain functional networks, improve motor performance, and optimize network flexibility. Accordingly, our study opens up new avenues for maximizing meaningful outcomes by promoting tailored noninvasive brain stimulation protocols for individual patients.

## Appendix A Supplementary data

Supplementary material Image 1
