# Supplementary material for: Disrupted functional network integrity and flexibility after stroke: Relation to motor impairments
Source: Neuroimage Clin. 2018 Jun 9;19:883–91. doi: 10.1016/j.nicl.2018.06.010 (PMC6008503; doi:10.1016/j.nicl.2018.06.010)
Supplement: Supplementary file 1 — Supplementary material [file mmc1.doc]

**Disrupted functional network integrity and flexibility after stroke: relation to motor impairments**

*Supplementary Material*

Image acquisition and preprocessing

Imaging was performed on a 3T TRIO scanner (Siemens, Erlangen, Germany) using a 12-channel head coil. All subjects underwent a single scanning session during which all functional images were collected using a T2*-weighted MRI transverse echo-planar images (EPI) with the following parameters: 130 functional volumes consisting of 48 axial slices; thickness/gap = 2.5 mm; matrix = 64 × 64; repetition time (TR) = 3250 ms; echo time (TE) = 30 ms; voxel size = 3 × 3 × 3 mm; flip angle (FA) = 90°; field of view (FOV) = 192 mm. The first six volumes were discarded to allow for T1 equilibrium effects, and data from the remaining 124 volumes were used in the analysis. A high resolution T1-weighted anatomical image (176 partitions; matrix = 256 × 240; TR = 7.92ms; TE = 2.48ms; 1.3 × 1.3 × 1.3 mm voxels; FA = 16°; FOV = 256 × 240 mm) and a field map (TE1 = 10 ms and TE2 = 12.46 ms, 3 × 3 × 2 mm resolution, 1 mm gap) were also acquired.

The data were preprocessed using Statistical Parametric Mapping 8 (SPM8; Wellcome Trust Centre for Neuroimaging, UK). For each subject, all functional images were realigned and unwarped to account for movement artefacts, co-registered to the subject’s structural image, normalized to the Montreal Neurological Institute echo planar imaging template (voxel size = 2 × 2 × 2 mm), and spatially smoothed using an 8 × 8 × 8 mm full width at half maximum Gaussian filter. No participants included in the current study showed motion correction that exceeded 4 mm or degrees on any axis.

Table S1. **Patient characteristics**. M=male; F=female; L=left; R=right; L/R=ambidextrous; CST=corticospinal tract; MCA=middle cerebral artery. Motor scores are provided as the average of a percentage of the score obtained with the unimpaired hand (i.e., %unaffected) across all three behavioral motor tests (nine hole peg test, box and block test, and grip strength).

| **Patient** | **Age**  **(years)** | **Sex** | **Affected**  **hand** | **Dominant**  **hand** | **Site of**  **lesion** | **Time since**  **stroke** | **Motor score  (%unaffected)** |
| --- | --- | --- | --- | --- | --- | --- | --- |
| 1 | 52 | M | L | R | R Insula | 5 months | 83.0 |
| 2 | 63 | F | L | R | R Pallidum | 2.1 months | 76.0 |
| 3 | 66 | M | L | R | R MCA | 7.1 years | 49.1 |
| 4 | 71 | M | R | R | L CST | 6.7 years | 10.4 |
| 5 | 55 | M | L | R | R MCA | 17.3 years | 85.7 |
| 6 | 52 | M | R | R | L MCA | 8 years | 58.3 |
| 7 | 56 | M | L | R | R CST | 2 months | 63.4 |
| 8 | 44 | M | R | R | L Putamen | 4.3 years | 13.2 |
| 9 | 53 | F | R | R | L CST | 3.9 months | 50.8 |
| 10 | 23 | M | L | R | R MCA | 4 months | 89.4 |
| 11 | 64 | M | L | R | R Thalamus | 1 month | 17.3 |
| 12 | 58 | M | L | R | R Putamen | 8.2 years | 70.3 |
| 13 | 30 | F | R | R | L Insula | 2.6 years | 69.4 |
| 14 | 53 | M | L | R | R MCA | 5 months | 4.49 |
| 15 | 48 | M | L | R | R Insula | 1.4 months | 67.0 |
| 16 | 50 | M | R | R | L MCA | 1 year | 7.96 |
| 17 | 64 | M | L | L/R | R MCA | 6.3 years | 33.4 |

Table S2. **Raw behavioral motor scores for the dominant hand (controls) and affected hand (stroke patients)**. Standard deviations in parentheses. BBT, Box and Block Test (number of blocks transferred in a minute); NHPT, Nine Hole Peg Test (pegs/s); grip strength (kg). ^a^ = Control > Stroke, p < 0.001.

| **Test** | **Control**  **Subjects** | **Stroke**  **Patients** |
| --- | --- | --- |
| BBT^a^ | 63.75 (11.5) | 27.06 (16.8) |
| NHPT^a^ | 0.74 (0.1) | 0.26 (0.24) |
| Grip strength^a^ | 82.87 (25.6) | 36.98 (29.4) |

Table S3. **Anatomical description for Network 1 (Dorsal Attention Network)**. Cluster volumes for the most extreme 5% of component loadings for Network 1, Montreal Neurological Institute (MNI) coordinates, and Brodmann area (BA) for the peak locations within each cluster.

| Cortical regions | Cluster volume | | | BAs for peak locations | | Peak MNI coordinates | | | | |
| --- | --- | --- | --- | --- | --- | --- | --- | --- | --- | --- |
|  | (mm^3^) | | (voxels) |  |  | *x* | | *y* | | *z* |
| Positive loadings |  | |  |  | |  | | | | |
| *Cluster 1: bilateral*  Precuneus  Superior parietal lobule  Anterior intraparietal sulcus  Cingulate gyrus, posterior division  Cingulate gyrus, anterior division  Postcentral gyrus  Central opercular cortex  Inferior frontal gyrus, pars opercularis    *Cluster 1: right hemisphere*  Lateral occipital cortex, superior division | 79,464 | | 9933 | | 7  7  7  23  24  2  43  44  39 | | 0  –30  –34  0  2  –54  –58  56  28 | | –46  –48  –40  –20  12  –22  –20  10  –74 | 60  60  44  44  38  40  18  4  34 |
| *Cluster 2: left hemisphere*  Lateral occipital cortex, inferior division  Cerebellum – Lobule VI | 10,672 | | 1334 | | 37  n/a | | –44  –26 | | –70  –60 | 2  –20 |
| *Cluster 3: right hemisphere*  Cerebellum – Lobule VI | 4736 | | 592 | | n/a | | 28 | | –60 | –20 |
| *Cluster 4: left hemisphere*  Lateral occipital cortex, superior division | 4144 | | 518 | | 39 | | –22 | | –76 | 32 |
| *Cluster 5: right hemisphere*  Putamen | | 1640 | 205 | | n/a | | 26 | | 0 | –10 |
| *Cluster 6: right hemisphere*  Thalamus | | 760 | 95 | | n/a | | 10 | | –16 | 6 |
| *Cluster 7: left hemisphere*  Primary visual cortex | 512 | | 64 | | 17 | | –14 | | –72 | 10 |

Table S4. **Anatomical description for Network 2 (Visual Network)**. Cluster volumes for the most extreme 5% of component loadings for Network 2, Montreal Neurological Institute (MNI) coordinates, and Brodmann area (BA) for the peak locations within each cluster.

| Cortical regions | Cluster volume | | BAs for peak locations | | Peak MNI coordinates | | | | |
| --- | --- | --- | --- | --- | --- | --- | --- | --- | --- |
|  | (mm^3^) | (voxels) |  |  | *x* | | *y* | | *z* |
| Positive loadings |  |  |  | |  | | | | |
| *Cluster 1: right hemisphere*  Lateral occipital cortex, superior division  Lateral occipital cortex, inferior division  Occipital fusiform gyrus  Primary visual cortex  Inferior temporal gyrus, temporooccipital part  Temporal occipital fusiform cortex | 30,552 | 3819 | | 19  18  18  17  19  19 | | 36  44  28  24  48  30 | | –84  –84  –86  –100  –56  –54 | 16  0  –10  –10  –16  –12 |
| *Cluster 2: left hemisphere*  Lateral occipital cortex, superior division  Lateral occipital cortex, inferior division  Occipital fusiform gyrus  Primary visual cortex  Inferior temporal gyrus, temporooccipital part  Temporal occipital fusiform cortex | 25,080 | 3135 | | 19  18  18  17  19  19 | | –30  –42  –28  –16  –52  –44 | | –88  –88  –86  –98  –60  ­–60 | 12  –4  –14  –14  –12  –14 |
| *Cluster 3: left hemisphere*  Middle frontal gyrus  Superior parietal lobule  Postcentral gyrus | 18,624 | 2328 | | 6  7  1 | | –36  –36  –46 | | –2  –42  –28 | 64  64  64 |
| *Cluster 4: right hemisphere*  Inferior temporal gyrus, posterior division  Inferior temporal gyrus, anterior division | 14,328 | 1791 | | 37  20 | | 52  52 | | –28  –4 | ­–20  –40 |
| *Cluster 5: left hemisphere*  Inferior temporal gyrus, posterior division  Inferior temporal gyrus, anterior division  Temporal fusiform cortex | 5792 | 724 | | 37  20  37 | | –50  –50  –36 | | –28  –10  –20 | –16  –38  –28 |
| *Cluster 6: right hemisphere*  Cerebellum – Lobule VI  Cerebellum – Lobule V | 4032 | 504 | | n/a  n/a | | 26  8 | | –54  –54 | –26  –14 |
| *Cluster 7: right hemisphere*  Superior parietal lobule | 1856 | 232 | | 7 | | 36 | | –44 | 66 |
| *Cluster 8: right hemisphere*  Precentral gyrus | 1408 | 176 | | 6 | | 36 | | –4 | 66 |

Table S5. **Anatomical description for Network 3 (Motor Network)**. Cluster volumes for the most extreme 5% of component loadings for Network 3, Montreal Neurological Institute (MNI) coordinates, and Brodmann area (BA) for the peak locations within each cluster.

| Cortical regions | Cluster volume | | BAs for peak locations | | Peak MNI coordinates | | | | |
| --- | --- | --- | --- | --- | --- | --- | --- | --- | --- |
|  | (mm^3^) | (voxels) |  |  | *x* | | *y* | | *z* |
| Positive loadings |  |  |  | |  | | | | |
| *Cluster 1: bilateral*  Supplementary motor cortex  Precentral gyrus  Precentral gyrus  Superior frontal gyrus  *Cluster 1: left hemisphere*  Postcentral gyrus  Postcentral gyrus  Postcentral gyrus  Superior parietal lobule  *Cluster 1: right hemisphere*  Precentral gyrus | 66,528 | 8316 | | 6  6  4  6  1  2  3  7  6 | | 0  –30  –2  18    –42  –40  –28  –34    52 | | –6  –10  –22  –6  –24  –38  –32  –48    4 | 62  62  52  68    58  56  56  58    38 |
| *Cluster 2: right hemisphere*  Superior parietal lobule  Lateral occipital cortex, superior division  Postcentral gyrus | 8392 | 1049 | | 7  7  2 | | 36  24  52 | | –38  –58  –20 | 52  52  42 |
| *Cluster 3: left hemisphere*  Precentral gyrus  Precentral gyrus | 2760 | 345 | | 6  44 | | –54  –54 | | 2  6 | 38  24 |
| Negative loadings |  |  | |  | |  | |  |  |
| *Cluster 1: bilateral*  Visual cortex  Primary visual cortex  Cerebellum – Crus I  Cerebellum – Vermis VI | 26,032 | 3254 | | 18  17  n/a  n/a | | 4  4  34  2 | | –84  –90  –64  –78 | 22  2  –26  –26 |

Table S6. **Anatomical description for Network 4 (Default-Mode Network)**. Cluster volumes for the most extreme 5% of component loadings for Network 4, Montreal Neurological Institute (MNI) coordinates, and Brodmann area (BA) for the peak locations within each cluster.

| Cortical regions | Cluster volume | | BAs for peak locations | | Peak MNI coordinates | | | | |
| --- | --- | --- | --- | --- | --- | --- | --- | --- | --- |
|  | (mm^3^) | (voxels) |  |  | *x* | | *y* | | *z* |
| Negative loadings |  |  |  | |  | | | | |
| *Cluster 1: bilateral*  Superior frontal gyrus  Dorsomedial prefrontal cortex  Ventromedial prefrontal cortex  Orbitofrontal cortex | 54,256 | 6782 | | 9  9  10  11 | | –10  –4  0  0 | | 38  58  54  44 | 54  28  6  –14 |
| *Cluster 2: bilateral*  Precuneus  Cingulate gyrus, posterior division | 17,400 | 2175 | | 7  23 | | 2  2 | | –56  –40 | 38  30 |
| *Cluster 3: right hemisphere*  Middle temporal gyrus, posterior division  Middle temporal gyrus, anterior division | 6488 | 811 | | 21  38 | | 56  56 | | –16  4 | –14  –30 |
| *Cluster 4: right hemisphere*  Lateral occipital cortex, superior division | 5632 | 704 | | 7 | | 56 | | –62 | 30 |
| *Cluster 5: left hemisphere*  Lateral occipital cortex, superior division | 4264 | 533 | | 7 | | –48 | | –66 | 30 |
| *Cluster 6: left hemisphere*  Middle temporal gyrus, posterior division  Middle temporal gyrus, anterior division | 4024 | 503 | | 21  38 | | –54  –54 | | –30  –6 | –10  –22 |
| *Cluster 7: right hemisphere*  Orbitofrontal cortex | 3248 | 406 | | 47 | | 38 | | 38 | –16 |
| *Cluster 8: left hemisphere*  Hippocampus | 2368 | 296 | | n/a | | –26 | | –18 | –18 |
| *Cluster 9: right hemisphere*  Hippocampus | 2176 | 272 | | n/a | | 28 | | –18 | –18 |
| *Cluster 10: left hemisphere*  Orbitofrontal cortex | 2024 | 253 | | 47 | | –36 | | 32 | –16 |

Figure S1: **Within-network regional activity differences**. The within-network analysis masked for the dominant 10% of component loadings for the Default-Mode Network (Network 4) revealed significantly reduced deactivity in bilateral precuneus in stroke patients relative to control subjects (p_corr_ < 0.05).

*
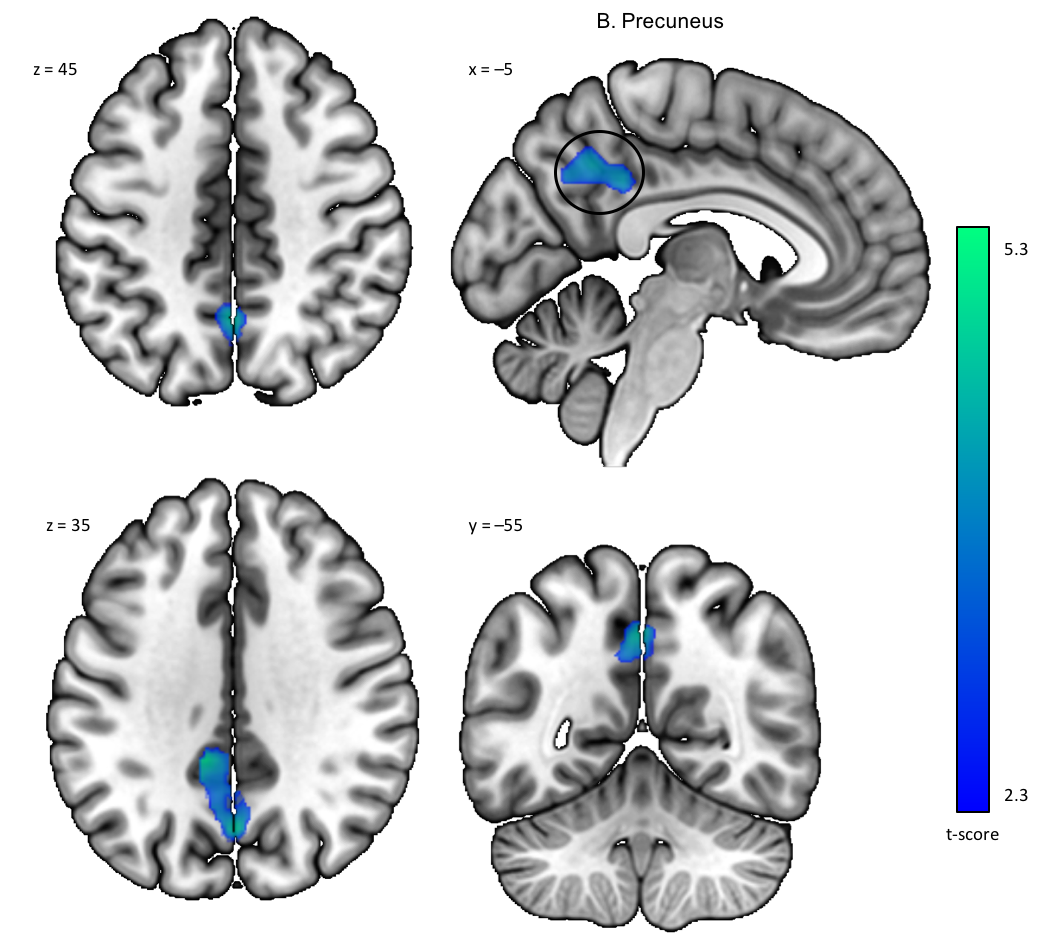
*
